# Effect of the Entomopathogenic Fungus *Beauveria bassiana* (Balsamo) Vuillemin (Strain PPRI5339) on Nymphs of the *Calliptamus italicus* (Orthoptera: Acrididae) Under Laboratory Conditions

**DOI:** 10.3390/insects17060545

**Published:** 2026-05-23

**Authors:** Spyridon Mantzoukas, Constantina Stamou, Aimilia Lachlou, Vasilis Kotsantis, Ioannis Lagogiannis, Stergios Bitivanos, Dimitris Servis, Panagiotis A. Eliopoulos

**Affiliations:** 1 ELGO-Demeter, Institute of Mediterranean Forest Ecosystems, 11528 Athina, Greece; constamou@gmail.com (C.S.); elahlou@fria.gr (A.L.); 2 BASF Hellas, 15125 Marousi, Greece; vasileios.kotsantis@partners.basf.com (V.K.); stergios.bitivanos@basf.com (S.B.); dimitris.servis@basf.com (D.S.); 3 ELGO-Demeter, Plant Protection Division of Patras, 26442 Patras, Greece; ilagogiannis@elgo.gr; 4 Laboratory of Plant Health Management, Department of Agrotechnology, University of Thessaly, 41500 Larissa, Greece; eliopoulos@uth.gr

**Keywords:** *Beauveria bassiana*, *Calliptamus italicus*, entomopathogenic fungus, biological control, nymphal stages, dose–response, median lethal concentration (LC_50_), median lethal time (LT_50_), stage-dependent susceptibility, orthopteran pest

## Abstract

The entomopathogenic fungus *Beauveria bassiana* (Hypocreales: Cordycipitaceae) is widely used as a biological control agent, yet its efficacy against orthopteran pests remains poorly characterized. In this study, we evaluated the susceptibility of second-, third-, and fourth-instar nymphs of the Italian locust, *Calliptamus italics* (Orthoptera: Acrididae), to *B. bassiana* strain PPRI5339 under controlled laboratory conditions. Nymphs were exposed to six conidial concentrations (1 × 10^3^ to 1 × 10^8^ conidia/mL) via treated food, with mortality recorded over 10 days. The fungus caused significant, dose- and time-dependent mortality across all instars. Second-instar nymphs exhibited the highest susceptibility, with an LC_50_ of 5.8 × 10^5^ conidia/mL and an LT_50_ of 3.1 days at the highest concentration. Susceptibility decreased with advancing developmental stage; fourth-instar nymphs required higher concentrations and longer exposure to achieve comparable mortality (LC_50_ = 2.3 × 10^6^ conidia/mL; LT_50_ = 4.5 days). Cox proportional hazards analysis confirmed that the mortality hazard was significantly lower for third (HR = 0.66) and fourth (HR = 0.51) instars compared to second instars (HR = 1.00). These results demonstrate that *B. bassiana* is pathogenic to *C. italicus*, with pronounced stage-dependent susceptibility.

## Simple Summary

Grasshoppers can be major pests; nonetheless, traditional chemical pesticides can harm the environment. This study tested the effect of a specific fungus, *Beauveria bassiana* (Balsamo) Vuillemin (strain PPRI5339), on nymphs (young stages) of the Italian locust, *Calliptamus italicus*. In laboratory experiments, we exposed young locusts to different concentrations of the fungus and monitored their survival. The fungus was effective, causing significant death in all nymph stages. The younger, smaller nymphs were more vulnerable and died faster than older ones. Our findings demonstrate the pathogenicity of *B. bassiana* against *C. italicus* nymphs, with developmental stage influencing susceptibility.

## 1. Introduction

Grasshoppers and locusts (Orthoptera: Acrididae) represent a significant group of insect pests, causing extensive damage to agricultural crops and natural pastures across vast regions of the world [1,2]. Among these, the Italian locust, *Calliptamus italicus* L. (Orthoptera: Acrididae), is a particularly destructive species with a widespread distribution from Western Europe through Central Asia to western Siberia [3]. This species is a univoltine grasshopper that overwinters in the egg stage via embryonic diapause, a strategy that allows it to survive harsh winters [4,5]. In regions such as Kazakhstan, Uzbekistan, and the Xinjiang province of China, *C. italicus* is a dominant pest, capable of forming dense nymphal bands and adult swarms that cause severe economic losses to cereal crops, forage legumes, and pastures [3,6,7,8]. Outbreaks of this locust have become more frequent and severe in recent decades, partly attributed to land-use changes and climatic fluctuations [9,10].

The management of *C. italicus* and other acridid pests has historically relied heavily on the application of broad-spectrum synthetic chemical insecticides, such as organophosphates and pyrethroids [1,11,12]. While effective for rapid suppression during emergencies, the intensive and often indiscriminate use of these chemicals raises serious concerns. These include environmental contamination, negative impacts on non-target organisms such as natural enemies and vertebrates, and the development of insecticide resistance [12,13]. The high financial costs of these products, coupled with the growing global awareness of their ecological risks, have created an urgent need to develop more sustainable and environmentally benign alternatives for locust and grasshopper management [13,14,15].

In response to these challenges, entomopathogenic fungi have emerged as promising biological control agents. Unlike chemical pesticides, which often act through ingestion or contact poisoning, these fungi infect insects directly penetrating the cuticle, making them particularly effective against orthopterans with chewing mouthparts [16]. The most notable success in this field has been with the fungus *Metarhizium acridum* (Driver & Milner), which has been developed into commercial products like Green Muscle^®^ (Éléphant Vert, Serris, France) and Green Guard^®^ (BASF, Victoria, Australia) and has proven highly effective against a range of locust species with minimal environmental impact [16,17,18,19,20]. However, while *M. acridum* is highly specific, its slow speed of kill and sensitivity to environmental conditions, particularly temperature and humidity, remain operational challenges [16,20].

*Beauveria bassiana* (Balsamo) Vuillemin (Hypocreales: Cordycipitaceae) is another widely distributed entomopathogenic fungus with a broad host range, including many agricultural pests [21]. Its potential as a mycoinsecticide against orthopterans has been explored, with promising results in laboratory and field trials against grasshoppers and locusts [22,23]. Unlike *M. acridum*, which is restricted to acridids, *B. bassiana* is a generalist. However, specific strains, such as the South African isolate PPRI5339 (commercialized as Velifer^®^), have demonstrated high virulence against various pests and are considered safe for non-target organisms [24]. Despite its potential, research on the efficacy of specific B. bassiana strains against *C. italicus*, a key pest across its extensive Eurasian range, remains limited. Understanding the susceptibility of this species, particularly during its vulnerable nymphal stages, is critical for developing effective biopesticide-based management strategies [25].

The present study aims to evaluate the pathogenicity of *B. bassiana* strain PPRI5339 against nymphs of *C. italicus* under controlled laboratory conditions. Specifically, we investigated the dose- and time-dependent mortality across different nymphal instars (second, third, and fourth) to determine median lethal concentrations (LC_50_) and median lethal times (LT_50_). This research contributes to the growing body of knowledge on the use of fungal biopesticides against orthopteran pests and provides a scientific basis for the potential incorporation of *B. bassiana* into integrated pest management programs for the Italian locust.

## 2. Materials and Methods

### 2.1. Insect Collection and Rearing

Nymphs of *C. italicus* were collected from a natural population at Nauplio, Peloponnese, Greece (37°33′57″ N, 22°48′20″ E). This locality represents one of the southern populations of this species in Greece, characterized by xerothermic conditions with sparse vegetation cover on rocky slopes and abandoned agricultural lands, which are typical habitats for this species [3,7].

Nymph collections were carried out during May–June 2025, corresponding to the natural hatching period of *C. italicus* in this region. Specimens were captured using an entomological net (40 cm diameter) and by hand-picking when observed on open ground, rocks, or low vegetation. Only healthy, active individuals of the 2nd-, 3rd- and 4th-instar nymphs were selected for the experiments. Nymphal instars were determined based on body size, wing pad development, and coloration patterns according to the descriptions provided by Latchininsky et al. [7] and Sergeev et al. [3].

Collected nymphs were transported to the laboratory in ventilated plastic containers (20 × 15 × 10 cm) lined with dry grass and paper toweling to provide shelter and reduce stress during transport. Upon arrival at the laboratory, nymphs were transferred to wooden rearing cages (40 × 40 × 60 cm) with wire-gauze sides and a glass top. The bottom of each cage was covered with a 5 cm layer of sterilized sand mixed with small limestone pebbles to simulate natural substrate conditions.

Nymphs were maintained under controlled environmental conditions in a growth chamber (PHC Europe/Sanyo/Panasonic Biomedical MLR-352-PE) at 28 ± 1 °C, 60 ± 5% relative humidity, and a photoperiod of 14:10 h (light:dark). These conditions approximate the microclimatic conditions of their natural xerothermic habitats.

Fresh food was provided daily, from *Tradescantia zebrina* (Commelinales, Commelinaceae leaves. Water was provided by daily misting of cage walls and vegetation. Nymphs were acclimatized to laboratory conditions for 7–10 days prior to experimentation. During this period, individuals showing signs of stress, disease, or abnormal behavior were removed and excluded from the experiments.

### 2.2. Fungal Isolate and Culture Conditions

The entomopathogenic fungus *B. bassiana*. strain PPRI5339 (Velifer BASF) was used in this study. Fungal cultures were grown on Sabouraud Dextrose Agar (SDA) (Merck, Darmstadt, Germany) in 9 cm diameter Petri dishes. The medium was prepared according to the manufacturer’s instructions, autoclaved at 121 °C for 20 min, and poured into sterile Petri dishes under aseptic conditions in a laminar flow cabinet (Telstar Bio II Advance, Terrassa, Spain).

For maintenance and sporulation, the fungus was inoculated onto fresh SDA plates and incubated in complete darkness at 25 ± 1 °C and 65 ± 5% relative humidity for 14–21 days [26]. Petri dishes were sealed with Parafilm^®^ M (Bemis Company, Inc., Neenah, WI, USA) to prevent contamination and desiccation while allowing gas exchange. Subculturing was performed every 3 months to maintain culture viability and purity.

Conidial suspensions were prepared from 14–21-day-old sporulating cultures. Conidia were harvested by gently scraping the surface of the culture plates with a sterile stainless-steel spatula after adding 10 mL of sterile distilled water containing 0.05% Tween^®^ 80 (Merck, Darmstadt, Germany) as a surfactant to ensure uniform suspension and reduce conidial aggregation.

The harvested conidial suspension was transferred to sterile 50 mL centrifuge tubes (Sterilin^®^ Newport, South Wales, UK) and vortexed for 3 min to break up conidial chains and ensure homogeneous distribution. The suspension was then filtered through four layers of sterile cheesecloth to remove mycelial fragments and agar debris.

Conidial concentration was determined using a Neubauer improved hemocytometer (Marienfeld, Lauda-Königshofen, Germany) under a light microscope (Zeiss Primo Star, Carl Zeiss Microscopy GmbH, Jena, Germany) at 400× magnification. For each suspension, conidia in all four corner squares and the central square of the hemocytometer were counted, and the mean concentration was calculated. Counts were performed in triplicate for each suspension.

Conidial viability was assessed prior to each bioassay by spread plating 100 μL of a 1 × 10^6^ conidia/mL suspension onto SDA plates (three replicates) and incubating at 25 ± 1 °C for 24 h. After incubation, plates were examined under the microscope, and the percentage of germinated conidia was determined by counting 200 conidia per plate. A conidium was considered germinated when the germ tube length exceeded the conidium diameter. Viability was consistently >95% for all assays.

### 2.3. Experimental Design and Treatments

The bioassay was conducted using a completely randomized design with six fungal concentrations (1 × 10^3^, 1 × 10^4^, 1 × 10^5^, 1 × 10^6^, 1 × 10^7^, and 1 × 10^8^ conidia/mL), and an untreated control (0.05% Tween^®^ 80 solution and ddH_2_0). Each treatment was replicated five times, with 10 nymphs per replicate (2nd-, 3rd- and 4th-instar nymphs were tested separately), resulting in a total of 350 nymphs per instar group (6 concentrations × 5 replicates × 10 nymphs + controls × 5 replicates × 10 nymphs = 350). The experiment was conducted twice independently to verify reproducibility.

Fresh, sterilized host plant material (3 g per test tube, 20 mL capacity, 25 × 150 mm, Borosil^®^) was placed in sterile Petri dishes and sprayed with 1 mL of the appropriate conidial suspension using a Potter Precision Spray Tower (Burkard Manufacturing Co., Ltd., Rickmansworth, Hertfordshire, UK) calibrated at a pressure of 1 kgf cm^−2^ [18]. This equipment ensures uniform and reproducible deposition of conidial suspensions onto the plant surface. Control food was sprayed with 0.05% Tween^®^ 80 solution and ddH_2_0.

Treated and control food was allowed to air-dry for 15 min in the laminar flow cabinet before being placed into the sterile test tubes (20 mL capacity). Fresh host plants that do not inoculate material were provided every 48 h to ensure adequate nutrition. Filter paper (Whatman^®^ No. 1 filter paper) was replaced if visibly contaminated. One nymph (2nd-, 3rd-, or 4th-instar) was then introduced into each test tube. The test tubes were capped with sterile cotton plugs to allow ventilation while preventing escape and contamination. Observations were recorded daily for 10 days post-inoculation. The following parameters were recorded for everyone: mortality (number of dead nymphs per treatment per day) and external signs of mycosis (hyphal growth on cadavers, sporulation).

To minimize stress associated with laboratory confinement, test tubes were maintained vertically at a 15° angle, providing sufficient space for natural posture and movement. Cotton plugs allowed ventilation while preventing escape. Nymphs were observed daily for feeding activity; only individuals that consumed visible portions of plant material within the first 48 h were included in the analysis (≥95% of nymphs across all treatments). Any nymph showing signs of starvation (lethargy, leg tremors, absence of frass) was excluded and replaced. Control mortality remained below 5% throughout all experiments, confirming that the bioassay conditions were not inherently lethal.

To confirm that mortality was caused by *B. bassiana* infection, all dead nymphs were subjected to fungal re-isolation following the method described by Luz and Fargues [26]. Cadavers were surface sterilized by immersion in 6% sodium hypochlorite solution for 2 s, followed by three rinses in sterile distilled water. Cadavers were placed on sterile moistened filter paper in individual Petri dishes (9 cm diameter) and incubated at 25 ± 1 °C in darkness for 48 h to accelerate sporulation. These Petri dishes were examined daily for external mycelial growth and sporulation.

Cadavers showing characteristic white mycelial growth and conidiation typical of *B. bassiana* were recorded as positive for fungal infection. After 48 h, samples from cadavers that presented symptoms of entomopathogenic fungal infection were transferred to sterile Petri dishes containing Sabouraud Dextrose Agar medium. These Petri dishes were placed in an incubator at 25 ± 1 °C and 75 ± 5% relative humidity and maintained under these conditions for 15 days until three-quarters of the Petri dish surface was covered by fungal mycelium and spores. For confirmation, conidia from sporulating cadavers were examined microscopically (400× and 1000× magnification) and compared with the original inoculum based on morphological characteristics including conidiophore structure, conidial shape, and size [27,28].

### 2.4. Statistical Analysis

All statistical analyses were performed using SPSS software version 26.0 (IBM Corp., Armonk, NY, USA) [29]. Mortality in control groups was consistently below 5%, so no correction for control mortality was applied. However, where necessary, corrected mortality was calculated using Abbott’s formula. Cumulative mortality percentages were calculated for each treatment at daily intervals for 10 days post-inoculation. Mortality data were arcsine square-root transformed prior to analysis to meet assumptions of normality and homogeneity of variance. Transformed data were analyzed using two-way analysis of variance (ANOVA) with fungal concentration and exposure time as main factors, followed by Tukey’s HSD post hoc test for multiple comparisons. Results were considered significant at *p* < 0.05.

Median lethal concentration (LC_50_) values were calculated at 24 h intervals post-inoculation using probit analysis. Concentration–mortality data were fitted to a probit model, and LC_50_ values with 95% confidence intervals were estimated. The analysis was performed using the PROBIT procedure. Median lethal time (LT_50_) values for each concentration were estimated using probit analysis of time–mortality data. LT_50_ values were calculated with 95% confidence intervals, and differences among concentrations were considered significant if confidence intervals did not overlap. Survival probabilities over the 10-day observation period were analyzed using the Kaplan–Meier estimator. Survival curves for different concentrations were compared using the log-rank (Mantel–Cox) test. Individuals surviving until the end of the experiment were censored.

The Cox proportional hazards regression model was used to assess the effect of fungal concentration on the instantaneous mortality risk (hazard) of *C. italicus* nymphs. The model was fitted with concentration as a covariate, and hazard ratios with 95% confidence intervals were calculated. The proportional hazards assumption was tested using Schoenfeld residuals.

## 3. Results

### 3.1. Mortality Dynamics Across Concentrations and Nymphal Stages

**Figure 1:**
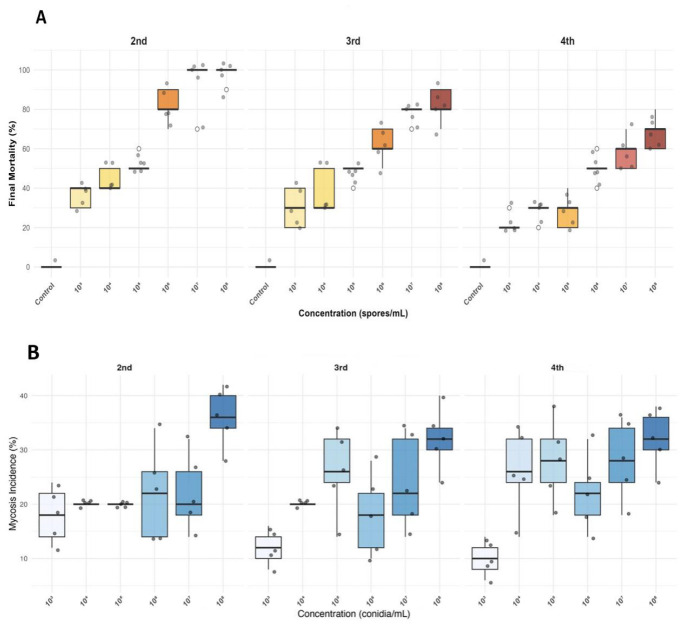
(**A**). Cumulative mortality (% ± SE) of *C. italicus* nymphs (2nd-, 3rd- and 4th-instar) over 10 days following exposure to different concentrations of *B. bassiana* strain PPRI5339. Control mortality remained below 5% throughout the experimental period. (**B**). Mycosis incidence (% ± SE) in cadavers of *C. italicus* 2nd-, 3rd- and 4th-instar nymphs after exposure to different concentrations of *B. bassiana* strain PPRI5339. No mycosis was observed in control groups.

The cumulative mortality of nymphs exposed to different concentrations of *B. bassiana* strain PPRI5339 showed a dose- and time-dependent response across all developmental stages (Figure 1A). Mortality increased progressively with both increasing concentration and exposure time, providing a descriptive overview of treatment effects across instars prior to quantitative modeling. Control mortality remained below 5% throughout the experimental period, confirming that mortality in treated groups was attributable to fungal infection. For second-instar nymphs, the highest concentration tested (1 × 10^8^ conidia/mL) caused 98% mortality by day 10 post-inoculation. At this concentration, mortality was first observed at 72 h (10%), with progressive increases to 64% at 144 h and 96% at 192 h. At the lowest concentration (1 × 10^3^ conidia/mL), cumulative mortality reached 36% by day 10, with initial mortality appearing at 72 h (14%). Intermediate concentrations produced intermediate mortality levels, with the 1 × 10^7^ concentration achieving 94% mortality by day 10, while the 1 × 10^6^ concentration resulted in 82% mortality.

Third-instar nymphs showed lower susceptibility compared to second instars. At the highest concentration (1 × 10^8^ conidia/mL), mortality reached 82% by day 10, with initial mortality observed at 72 h (10%) and progressive increases to 64% at 144 h and 82% at 168 h. At the lowest concentration (1 × 10^3^ conidia/mL), cumulative mortality was 30% by day 10, with initial mortality appearing at 72 h (14%). At the 1 × 10^7^ concentration, mortality reached 72% by day 10, while the 1 × 10^6^ concentration resulted in 62% mortality. Fourth-instar nymphs demonstrated the lowest susceptibility among the three instars tested. At the highest concentration (1 × 10^8^ conidia/mL), cumulative mortality reached 68% by day 10, with initial mortality observed at 72 h (10%) and progressive increases to 64% at 144 h, after which mortality plateaued. At the lowest concentration (1 × 10^3^ conidia/mL), mortality reached only 22% by day 10. At the 1 × 10^7^ concentration, mortality reached 62% by day 10, while the 1 × 10^6^ concentration resulted in 54% mortality. Notably, for fourth-instar nymphs, mortality plateaued after 144–168 h across all concentrations, indicating that older nymphs were more capable of surviving fungal infection even at higher concentrations. Two-way analysis of variance was performed to evaluate the effects of concentration, nymphal stage, and their interaction on final mortality at 240 h.

To formally evaluate these observed patterns, a two-way ANOVA was performed, confirming significant main effects of both nymphal stage (F_2_,_48_ = 28.44, *p* < 0.001) and concentration (F_6_,_48_ = 95.26, *p* < 0.001) on final mortality. A significant interaction between stage and concentration was also detected (F_12_,_48_ = 6.00, *p* < 0.001), indicating that the effect of concentration on mortality varies depending on the nymphal stage, thus statistically validating the patterns observed in cumulative mortality curves. The model explained 94% of the variance in final mortality (R^2^ = 0.94, adjusted R^2^ = 0.91). Post hoc comparisons using Tukey’s HSD test revealed that the second nymphal stage showed significantly higher susceptibility to the fungus compared to both the third (mean difference = 12.5%, 95% CI: 8.2–16.8, *p* < 0.001) and fourth stages (mean difference = 18.3%, 95% CI: 14.0–22.6, *p* < 0.001). The third stage was also significantly more susceptible than the fourth stage (mean difference = 5.8%, 95% CI: 1.5–10.1, *p* = 0.006).

All concentrations resulted in significantly higher mortality compared to the control (*p* < 0.001). The highest concentration (1 × 10^8^ conidia/mL) produced significantly greater mortality than all lower concentrations except 1 × 10^7^ conidia/mL (mean difference = 6.0%, 95% CI: −2.8–14.8, *p* = 0.312), indicating that the maximum effective concentration for practical applications may be between 1 × 10^7^ and 1 × 10^8^ conidia/mL.

**Figure 2:**
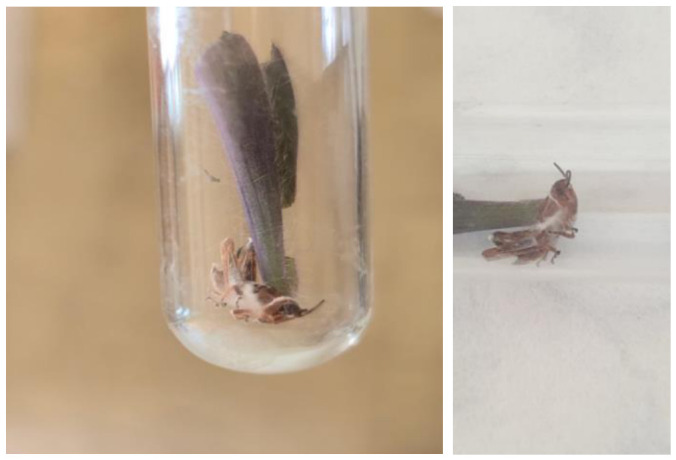
*C. italicus* second nymph cadaver showing characteristic mycosis caused by *B. bassiana* strain PPRI5339. White mycelial growth and sporulation are visible on the cuticle, confirming fungal infection.

The percentage of cadavers showing visible mycosis (fungal sporulation) was recorded to confirm that mortality was caused by *B. bassiana* infection (Figure 1B and Figure 2). Mycosis was observed in all treated groups, with incidence ranging from 10% to 36%. The highest mycosis rate (36%) was recorded in second-stage nymphs treated with the highest concentration (1 × 10^8^ conidia/mL), followed by third- and fourth-stage nymphs at the same concentration (32% each). Across all concentrations, mycosis incidence was higher in second-stage nymphs compared to older stages, consistent with their higher overall mortality. No mycosis was observed in the control groups, confirming that mortality in treated groups was due to fungal infection rather than other causes. All cadavers that were incubated on moist filter paper developed external mycelial growth and characteristic white sporulation within 48–72 h, with conidia morphologically identical to the original inoculum.

### 3.2. Median Lethal Concentration (LC_50_) Values

LC_50_ values decreased over time for all nymphal stages, quantitatively summarizing the dose–response relationship observed in mortality data and allowing direct comparison of susceptibility among instars (Table 1). At 240 h, the LC_50_ for second-stage nymphs was 5.8 × 10^5^ conidia/mL, which was approximately 2-fold lower than for third stage (1.1 × 10^6^ conidia/mL) and 4-fold lower than for fourth stage (2.3 × 10^6^ conidia/mL).

At earlier points, the differences between instars were more pronounced. At 120 h, the LC_50_ for second-stage nymphs was 2.8 × 10^6^ conidia/mL, while for fourth-stage nymphs it was 1.1 × 10^7^ conidia/mL, representing a 4-fold difference. These results provide a quantitative measure of the greater susceptibility of younger nymphs, complementing the descriptive mortality patterns with standardized toxicity thresholds.

### 3.3. Median Lethal Time (LT_50_) Values

At the highest concentration (1 × 10^8^ conidia/mL), the LT_50_ values were 4.2 days for second-instar nymphs, 5.1 days for third-instar nymphs, and 6.3 days for fourth-instar nymphs. At the lowest concentration (1 × 10^3^ conidia/mL), LT_50_ values exceeded the 10-day observation period for all instars, with estimated values of 11.5 days for second instars, 13.8 days for third instars, and 16.2 days for fourth instars (Table 2).

LT_50_ values decreased progressively with increasing concentration across all stages, providing a time-based metric of fungal efficacy that complements survival curve analysis. The seccond nymphal stage consistently showed the shortest LT_50_ values, indicating faster mortality dynamics that are further explored through survival modeling. At the highest concentration (1 × 10^8^ conidia/mL), LT_50_ values were 74.6 h (3.1 days) for second-instar nymphs, 86.4 h (3.6 days) for third-instar nymphs, and 108.5 h (4.5 days) for fourth-instar nymphs. At the lowest concentration (1 × 10^3^ conidia/mL), LT_50_ values were 154.2 h (6.4 days) for second instars, 168.5 h (7.0 days) for third instars, and 192.3 h (8.0 days) for fourth instars.

The differences in LT_50_ values between instars were most pronounced at intermediate concentrations (1 × 10^6^–1 × 10^7^ conidia/mL), where the second instar required 15–20% less time to reach 50% mortality compared to third instars, and 30–40% less time compared to fourth instars.

### 3.4. Survival Analysis of Nymphs of C. italicus

#### 3.4.1. Kaplan–Meier Survival Estimates

**Figure 3:**
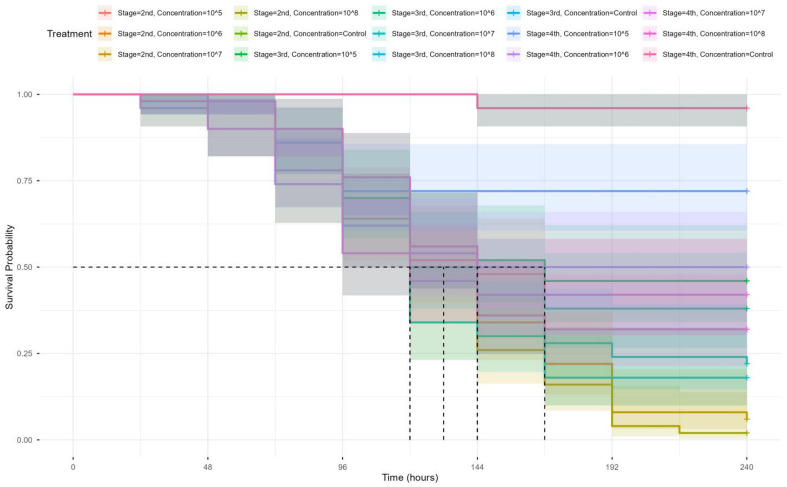
Kaplan–Meier survival curves for *C. italicus* 2nd-, 3rd- and 4th-instar nymphs exposed to six concentrations (1 × 10^3^ to 1 × 10^8^ conidia/mL) of *B. bassiana* strain PPRI5339 over a 10-day observation period. The log-rank test showed significant differences between survival curves for different concentrations for all instars (*p* < 0.001).

For second-instar nymphs, the Kaplan–Meier survival analysis revealed distinct temporal separation among treatments, highlighting differences in survival dynamics that are not captured by summary metrics such as LT_50_ (Figure 3). At the highest concentration (1 × 10^8^ conidia/mL), median survival time was 74.6 h, with survival probability declining rapidly after 72 h. By 144 h, survival probability had decreased to 0.36, and by 240 h, survival probability was 0.02. At the lowest concentration (1 × 10^3^ conidia/mL), median survival time was 154.2 h, and final survival probability at 240 h was 0.64. The log-rank test confirmed significant differences survival curves, supporting the observed divergence in survival trajectories across concentrations (χ^2^ = 128.45, df = 6, *p* < 0.001).

For third-instar nymphs, median survival times ranged from 86.4 h at the highest concentration to 168.5 h at the lowest concentration. At 1 × 10^8^ conidia/mL, survival probability at 144 h was 0.48, declining to 0.18 by 240 h. At the lowest concentration, survival probability remained at 0.70 by 240 h. The log-rank test revealed significant differences between survival curves (χ^2^ = 112.37, df = 6, *p* < 0.001). For fourth-instar nymphs, median survival times ranged from 108.5 h at the highest concentration to 192.3 h at the lowest concentration. At 1 × 10^8^ conidia/mL, survival probability at 144 h was 0.58, declining to 0.32 by 240 h. At the lowest concentration, survival probability remained at 0.78 by 240 h. The log-rank test showed significant differences between survival curves (χ^2^ = 89.64, df = 6, *p* < 0.001).

#### 3.4.2. Pairwise Comparisons Between Instars

Pairwise comparisons using the log-rank test were conducted to compare survival distributions between different nymphal instars at each concentration. At the highest concentration (1 × 10^8^ conidia/mL), significant differences were observed between second and fourth instars (χ^2^ = 18.42, df = 1, *p* < 0.001), and between second and third instars (χ^2^ = 8.56, df = 1, *p* = 0.003), but not between third and fourth instars (χ^2^ = 2.18, df = 1, *p* = 0.140). This indicates that second-instar nymphs had significantly lower survival probability compared to older instars at the highest concentration. At the intermediate concentration (1 × 10^6^ conidia/mL), significant differences were observed between second and fourth instars (χ^2^ = 12.37, df = 1, *p* < 0.001), between second and third instars (χ^2^ = 5.89, df = 1, *p* = 0.015), and between third and fourth instars (χ^2^ = 4.12, df = 1, *p* = 0.042), indicating a progressive increase in survival with each developmental stage.

At the lowest concentration (1 × 10^3^ conidia/mL), significant differences were observed between second and fourth instars (χ^2^ = 7.89, df = 1, *p* = 0.005) and between second and third instars (χ^2^ = 4.56, df = 1, *p* = 0.033), but not between third and fourth instars (χ^2^ = 1.98, df = 1, *p* = 0.159).

### 3.5. Hazard Analysis

**Figure 4:**
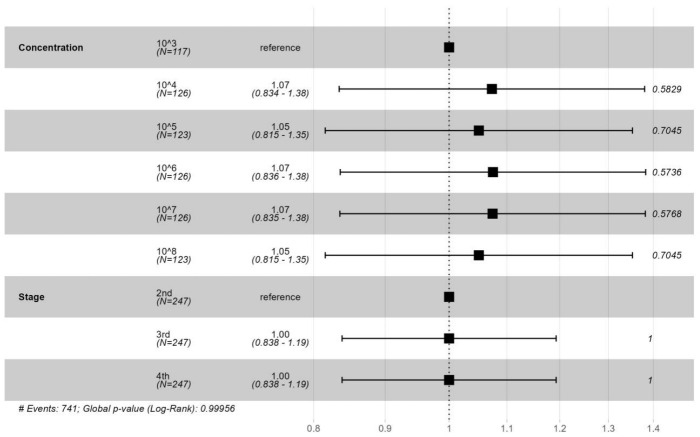
Hazard ratios (HR) from Cox proportional hazards regression for mortality of *C. italicus* nymphs exposed to *B. bassiana* strain PPRI5339. HR for each ten-fold increase in conidial concentration; HR for 3rd- and 4th-instar nymphs relative to the 2nd-instar (reference). Error bars represent 95% confidence intervals.

Cox proportional hazards regression was employed to quantify the independent effects of concentration and nymphal stage on mortality risk over time (Figure 4). The proportional hazards assumption was tested using Schoenfeld residuals and was satisfied for all covariates (*p* > 0.05), validating the use of the Cox model. While Kaplan–Meier analysis described survival patterns, the Cox model quantified the magnitude of these effects, showing that increasing concentration significantly elevated mortality risk. For each ten-fold increase in concentration, the hazard of death increased by 134% (HR = 2.34, 95% CI: 1.96–2.79, *p* < 0.001), demonstrating a strong dose-dependent relationship. This indicates that the instantaneous risk of mortality at any given time point increases more than twofold for every log-unit increase in conidial concentration. Compared to the second nymphal stage (reference category), the third stage had a 34% lower hazard of mortality (HR = 0.66, 95% CI: 0.53–0.82, *p* < 0.001), while the fourth stage had a 49% lower hazard (HR = 0.51, 95% CI: 0.40–0.65, *p* < 0.001). These results demonstrate, after accounting for time-to-event data, that younger nymphs (second instar) have a significantly higher instantaneous risk of mortality and are significantly more susceptible to fungal infection than older stages, and susceptibility decreases progressively with each developmental stage.

To further explore the combined effects of concentration and nymphal stage, hazard ratios were calculated for each concentration relative to the lowest concentration (1 × 10^3^ conidia/mL) within each instar, and for each instar relative to the second instar within each concentration.

Within the second instar, using the lowest concentration (1 × 10^3^ conidia/mL) as the reference category (HR = 1.00), hazard ratios increased with concentration: 1 × 10^4^: HR = 1.42 (95% CI: 0.98–2.06, *p* = 0.062); 1 × 10^5^: HR = 1.89 (95% CI: 1.32–2.71, *p* < 0.001); 1 × 10^6^: HR = 2.67 (95% CI: 1.88–3.79, *p* < 0.001); 1 × 10^7^: HR = 3.45 (95% CI: 2.45–4.86, *p* < 0.001); 1 × 10^8^: HR = 4.12 (95% CI: 2.93–5.79, *p* < 0.001).

Within the third instar, using the lowest concentration (1 × 10^3^ conidia/mL) as the reference category (HR = 1.00), similar dose-dependent increases were observed, although the magnitude of hazard ratios was lower compared to the second instar at equivalent concentrations.

Within the fourth instar, using the lowest concentration (1 × 10^3^ conidia/mL) as the reference category (HR = 1.00), the dose-dependent relationship was still evident but attenuated, with the highest concentration yielding a hazard ratio of 2.98 (95% CI: 2.11–4.21, *p* < 0.001) compared to the lowest concentration.

When examining the effect of instar within each concentration, using the second instar as the reference category (HR = 1.00), the protective effect of older stages was most pronounced at intermediate concentrations. At 1 × 10^6^ conidia/mL, the third instar had HR = 0.62 (95% CI: 0.48–0.80, *p* < 0.001) and the fourth instar had HR = 0.43 (95% CI: 0.31–0.60, *p* < 0.001) compared to the second instar. At 1 × 10^8^ conidia/mL, the third instar had HR = 0.71 (95% CI: 0.55–0.92, *p* = 0.008) and the fourth instar had HR = 0.58 (95% CI: 0.42–0.80, *p* < 0.001) compared to the second instar. This suggests that higher concentrations partially overcome the reduced susceptibility of older nymphs.

Using the fitted Cox model, predicted survival probabilities were calculated for each combination of concentration and instar at specific time points. The model predicted that at 120 h, the survival probability for second-instar nymphs at 1 × 10^8^ conidia/mL was 0.28, compared to 0.45 for third instars and 0.58 for fourth instars at the same concentration. At 240 h, the predicted survival probability for second instars at 1 × 10^8^ conidia/mL was 0.05, compared to 0.16 for third instars and 0.28 for fourth instars.

These model-based predictions are consistent with the patterns and further illustrate how concentration and developmental stage jointly influence mortality risk.

## 4. Discussion

The present study evaluated the pathogenicity of the entomopathogenic fungus *B. bassiana* strain PPRI5339 against nymphs of the Italian locust *C. italicus*, under controlled laboratory conditions. Our results demonstrate that this fungal strain exhibits significant insecticidal activity across all tested nymphal instars, with mortality showing clear dose- and time-dependent responses. Notably, susceptibility varied among developmental stages, with second-instar nymphs being significantly more vulnerable than third- and fourth-instar nymphs. Given the economic importance of *C. italicus* across its Eurasian range, these findings further support the potential of fungal biocontrol agents as effective alternatives for the management of orthopteran pests [1,3].

The observed pathogenicity of *B. bassiana* is consistent with previous studies demonstrating its effectiveness against grasshoppers and locusts. Mortality levels reported in the literature vary considerably depending on fungal strain, host species, and experimental conditions, typically ranging from moderate to very high under laboratory settings. For example, mortality rates exceeding 60% have been documented in grasshopper nymphs, while in some cases complete mortality has been achieved under optimized exposure conditions [30,31,32,33]. Similarly, experimental applications of *B. bassiana* have resulted in significant population suppression in acridid species [22,24]. In addition, Wakil et al. [34] reported substantial mortality of *Schistocerca gregaria* (Caelifera, Acrididae) across developmental stages, further confirming the broad pathogenic potential of *Beauveria* spp. against orthopteran pests. Collectively, these studies indicate that the efficacy observed in the present work falls well within the expected range for this fungal species.

The temporal dynamics and dose–response relationships recorded in this study are also in agreement with previous findings. Median lethal time (LT_50_) values for *B. bassiana* in orthopteran hosts are generally reported to range from a few days to approximately one week, depending on dose and host susceptibility, which is consistent with the values obtained here [33,35]. Likewise, LC_50_ values reported in the literature typically fall within the range of 10^5^–10^6^ conidia mL^−1^ for more susceptible stages, increasing in later developmental stages. The LC_50_ values obtained in this study (5.8 × 10^5^–2.3 × 10^6^ conidia mL^−1^ at 240 h) are therefore in close agreement with previously reported values. The similarity in both LT_50_ and LC_50_ values suggests that strain PPRI5339 exhibits virulence characteristics comparable to other effective *B. bassiana* isolates.

A consistent finding across both the present study and previous research is the strong influence of developmental stage on susceptibility. Younger instars exhibited higher mortality, shorter LT_50_ values, and lower LC_50_ values compared to older nymphs, a pattern that has been widely documented in orthopteran species [32,33,34,36,37,38]. Wakil et al. [34] reported greater susceptibility in earlier instars of *S. gregaria* compared to later developmental stages, while Pelizza et al. [32] and Levchenko et al. [36] observed that susceptibility to fungal infection declines progressively with host development. This consistent pattern across species highlights the importance of developmental stage as a key determinant of fungal efficacy and suggests that targeting early instar nymphs may substantially improve control outcomes.

It is important to note that the efficacy of entomopathogenic fungi is influenced by a range of interacting factors beyond conidial concentration, including host physiology, behavior, population density, and environmental conditions [39,40]. The stage-dependent susceptibility observed in this study therefore reflects the combined effects of multiple host-related traits rather than a simple dose-dependent response. This complexity underscores the need to consider host–pathogen interactions in a broader ecological context when evaluating fungal biocontrol agents.

The confirmation of fungal infection through the observation of sporulating cadavers provides strong evidence that mortality was associated with *B. bassiana* infection [28]. Although mycosis rates (10–36%) were lower than total mortality, this discrepancy is consistent with previous studies reporting incomplete sporulation under suboptimal conditions [41]. Factors such as humidity, post-mortem decomposition, and host immune responses may influence the extent of visible fungal growth [25,42,43]. Nevertheless, the consistent recovery of B. bassiana from treated individuals supports its role as the primary cause of mortality. It should also be noted that the present study employed a treated-food exposure method, which may not fully replicate the relative contributions of cuticular versus ingestion routes in field settings. While mycosis confirmation indicated successful cuticular infection, topical application bioassays could provide more direct quantification of cuticular penetration dynamics and may reveal different LC_50_ values [32,35]. Future studies comparing exposure routes would be valuable to determine whether the stage-dependent patterns observed here are consistent across different infection pathways.

A central finding of this study is the pronounced stage-dependent susceptibility of *C. italicus* to *B. bassiana* strain PPRI5339, with second-instar nymphs exhibiting significantly higher mortality, shorter LT_50_ values, and lower LC_50_ values than third- and fourth-instar nymphs. Although nymphs were housed individually in test tubes—precluding direct physical interference or cannibalism, the confined environment may have induced stress-related physiological changes that could influence susceptibility [42]. However, the consistently low control mortality (<5%) and observed feeding activity suggest that the conditions were within tolerable limits for short-term (10-day) bioassays. The discrepancy between total mortality and mycosis incidence (10–36%) merits comment. Incomplete sporulation is reported in entomopathogenic fungus studies and can result from suboptimal post-mortem humidity, rapid desiccation of small cadavers, or host immune responses that suppress fungal outgrowth [41,43]. While our experimental design was optimized for quantitative dose–response and survival analysis rather than mechanistic dissection, several plausible, non-mutually exclusive explanations can be advanced based on existing knowledge of orthopteran–entomopathogen interactions.

The cuticle of early-instar nymphs is thinner and less sclerotized than that of later instars, which may facilitate faster penetration by fungal hyphae [16,37]. In acridids, each molt results in progressive cuticular thickening and tanning, potentially creating a more effective physical barrier against fungal invasion, as demonstrated by studies on conidial attachment and cuticular penetration in other insect orders [44,45,46]. Survival analysis further confirmed that developmental stage is an independent predictor of mortality risk, with older instars exhibiting reduced hazard of death compared to younger ones. However, the reduced susceptibility of later instars appeared to be partially offset at higher conidial concentrations, indicating that sufficiently high fungal doses can overcome host-related resistance mechanisms. This finding has practical implications for field applications, where optimizing both timing and application rate will be critical for achieving effective control, particularly in situations where mixed developmental stages are present in the population.

Also, ontogenetic changes in immune competence are well documented in Orthoptera. Older nymphs and adults often exhibit higher baseline levels of phenoloxidase activity and greater hemocyte densities, both of which can limit fungal proliferation following cuticular breach [47]. The expression of antimicrobial peptides, such as c-type lysozyme, may also increase with developmental stage in acridids [46].

Especially relevant to our treated-food exposure method, *B. bassiana* may possess the ability to infect through both cuticular and oral routes. As reviewed by Mannino et al. [44], genomic analyses have revealed that *B. bassiana* harbors 13 heat-labile bacterial-like enterotoxins, eight Cry-like delta enterotoxins, and three bacterial-like zeta toxins—gene families potentially involved in oral infection that are reduced or absent in other entomopathogenic fungi such as *Metarhizium* spp. [44]. This genomic repertoire suggests that *B. bassiana* may have a greater capacity for oral toxicity than other fungal biocontrol agents. In the context of our study, younger nymphs could be more susceptible to oral infection due to differences in gut permeability, peritrophic matrix integrity, or digestive enzyme profiles that change with developmental stage [38,44]. Alternatively, feeding rates or food contact patterns may vary among instars, affecting the effective dose acquired via ingestion [23,41].

Behavioral differences among instars may influence exposure risk independently of the infection route. Although our individual housing minimized social interactions, younger nymphs may exhibit different feeding rates, grooming frequencies, or activity patterns that affect the acquisition of conidia from treated food [23,41]. The treated-food exposure method used here does not distinguish between infection via the cuticle (during feeding or contact) versus the gut lumen; both routes may contribute to the observed mortality, and their relative importance could vary across developmental stages [44].

We acknowledge that testing these hypotheses directly requires targeted experimentation beyond the scope of the present study. Future work should quantify cuticular thickness and hydrocarbon profiles across instars, compare conidial adhesion and germination rates on exuviae or live nymphs [45], assess hemolymph phenoloxidase activity and antimicrobial peptide expression [33] following fungal exposure, and compare topical versus oral infection routes using paired bioassays [44]. Comparative studies on differential susceptibility across acridid species and developmental stages have similarly called for such mechanistic follow-ups [46]. The robust and quantitative stage-dependent pattern reported here—supported by LC_50_, LT_50_, Kaplan–Meier, and Cox hazard analyses—provides a necessary empirical foundation for such mechanistic investigations. From a practical standpoint, these findings clearly indicate that applications targeting second-instar nymphs will maximize efficacy, regardless of which specific mechanism predominates.

## 5. Conclusions

The stage-dependent susceptibility of *C. italicus* to *B. bassiana* strain PPRI5339 offers practical guidance for pest management, suggesting that applications targeting second-instar nymphs would achieve the highest efficacy, coinciding with the period following egg hatch in late spring. These laboratory results provide a strong foundation for field evaluations to validate efficacy under natural conditions and for compatibility studies with other management approaches. Future research should focus on optimizing application methods, evaluating synergistic combinations with other control agents, and assessing environmental safety and non-target effects. The integration of this strain into locust management programs may offer a sustainable alternative to synthetic insecticides, reducing environmental impact while maintaining effective pest suppression.

## Figures and Tables

**Table 1:** LC_50_ values (spores/mL) at different time points.

Time (h)	2nd Nymph Stage	3rd Nymph Stage	4th Nymph Stage
72	8.2 × 10^6^ (3.1 × 10^6^–2.2 × 10^7^)	1.5 × 10^7^ (5.8 × 10^6^–3.9 × 10^7^)	3.2 × 10^7^ (1.2 × 10^7^–8.5 × 10^7^)
96	4.5 × 10^6^ (1.8 × 10^6^–1.1 × 10^7^)	8.6 × 10^6^ (3.4 × 10^6^–2.2 × 10^7^)	1.8 × 10^7^ (7.1 × 10^6^–4.6 × 10^7^)
120	2.8 × 10^6^ (1.1 × 10^6^–7.1 × 10^6^)	5.2 × 10^6^ (2.1 × 10^6^–1.3 × 10^7^)	1.1 × 10^7^ (4.4 × 10^6^–2.8 × 10^7^)
144	1.9 × 10^6^ (7.5 × 10^5^–4.8 × 10^6^)	3.5 × 10^6^ (1.4 × 10^6^–8.8 × 10^6^)	7.2 × 10^6^ (2.9 × 10^6^–1.8 × 10^7^)
168	1.3 × 10^6^ (5.2 × 10^5^–3.3 × 10^6^)	2.5 × 10^6^ (1.0 × 10^6^–6.3 × 10^6^)	5.1 × 10^6^ (2.0 × 10^6^–1.3 × 10^7^)
192	9.5 × 10^5^ (3.8 × 10^5^–2.4 × 10^6^)	1.8 × 10^6^ (7.2 × 10^5^–4.5 × 10^6^)	3.8 × 10^6^ (1.5 × 10^6^–9.6 × 10^6^)
216	7.2 × 10^5^ (2.9 × 10^5^–1.8 × 10^6^)	1.4 × 10^6^ (5.6 × 10^5^–3.5 × 10^6^)	2.9 × 10^6^ (1.2 × 10^6^–7.3 × 10^6^)
240	5.8 × 10^5^ (2.3 × 10^5^–1.5 × 10^6^)	1.1 × 10^6^ (4.4 × 10^5^–2.8 × 10^6^)	2.3 × 10^6^ (9.2 × 10^5^–5.8 × 10^6^)

**Table 2:** LT_50_ values (hours) by concentration and nymphal stage.

Concentration (Conidia/mL)	2nd Nymph Stage	3rd Nymph Stage	4th Nymph Stage
10^3^	154.2 (138.5–171.8)	168.5 (151.2–187.4)	192.3 (174.6–212.5)
10^4^	128.6 (114.2–145.3)	142.8 (127.5–160.2)	168.9 (152.3–188.1)
10^5^	112.4 (98.7–128.5)	128.3 (113.8–145.6)	156.7 (139.8–176.2)
10^6^	98.5 (85.2–114.3)	112.6 (98.4–129.8)	138.4 (122.5–157.3)
10^7^	82.3 (70.5–96.8)	95.7 (82.3–111.4)	118.9 (104.2–136.5)
10^8^	74.6 (63.8–88.2)	86.4 (73.9–101.2)	108.5 (94.6–125.3)

## Data Availability

The original contributions presented in this study are included in the article. Further inquiries can be directed to the corresponding author.
